# Admission characteristics of patients with short term hospitalization

**DOI:** 10.1186/s13584-024-00639-3

**Published:** 2024-09-26

**Authors:** Yael Frenkel Nir, Yuval Levy, Ehud Grossman, Eyal Klang

**Affiliations:** 1 Medical management department, The Chaim Sheba Medical Center, Tel Hashomer, Israel; 2 Faculty of Medicine, Tel Aviv University, Tel Aviv, Israel; 3 Adelsom School of Medicine, Ariel University, Ariel, Israel; 4 Department of Diagnostic Imaging, Sheba Medical Center, Tel Hashomer, Israel

**Keywords:** Short hospitalization, Emergency department, Medical wards, Admission diagnosis, Short stay unit

## Abstract

**Background:**

Sheba Medical Center (SMC) is the largest hospital in Israel and has been coping with a steady increase in total Emergency Department (ED) visits. Over 140,000 patients arrive at the SMC's ED every year. Of those, 19% are admitted to the medical wards. Some are very short hospitalizations (one night or less). This puts a heavy burden on the medical wards. We aimed to identify the characteristics of short hospitalizations.

**Methods:**

We retrospectively retrieved data of consecutive adult patients admitted to our hospital during January 1, 2013, to December 31, 2019. We limited the cohort to patients who were admitted to the medical wards. We divided the study group into those with short, those with non-short hospitalization and those who were discharged from the ED.

**Results:**

Out of 133,126 admissions, 59,994 (45.0%) were hospitalized for short term. Patients in the short hospitalization group were younger and had fewer comorbidities. The highest rate of short hospitalization was recorded during night shifts (58.4%) and the rate of short hospitalization was associated with the ED daily patient load (r = 0.35, *p* < 0.001). The likelihood of having a short hospitalization was most prominent in patients with suicide attempt (80.0% of those admitted for this complaint had a short hospitalization), followed by hypertension (68.6%). However, these complaints accounted for only 0.7% of the total number of short hospitalizations. Cardiac and neurological complaints however, made up 27.4% of the short hospitalizations. The 30-days mortality rate was 7.0% in the non-short hospitalization group, 4.3% in the short hospitalization group and 0.9% in those who were discharged from the ED.

**Conclusions:**

Short hospitalizations in medical wards have special characteristics that may render them predictable. Increasing the rate of treating personnel per patient during peak hours and referring subsets of patients with cardiac and neurological complaints to ED-associated short term observation units may decrease short admissions to medical departments.

**Supplementary Information:**

The online version contains supplementary material available at 10.1186/s13584-024-00639-3.

## Introduction

Health systems worldwide are under pressure due to a shortage of hospital beds. The demand for in-hospital care is rising, while the number of available beds remains constant. Israel (like many other countries around the globe) is coping with a steady increase in total emergency department (ED) visits, coupled by a steady reduction in hospitalization rates. [1]

Emergency departments and in-hospital care have to deal with overflow of patients. Decreasing the rate of preventable admissions may reduce the burden on the medical wards and increase the quality of care given to those patients who really need in-hospital care.

Very short admissions are defined as conditions that can be managed with timely and effective treatment in the outpatient setting [2]. Identifying these potentially very short admissions has always been a big challenge. Previous studies were unable to identify objective factors associated with potentially preventable admissions [3–8]. Many of the hospitalizations are of short-term. Whether short hospitalizations are associated with inappropriate admissions and are preventable, is controversial. Despite this controversy, various studies evaluated the characteristics of short hospitalizations to identify potential preventable admissions. Pope et al. demonstrated, in a cross-sectional analysis, that admissions in the middle of the week or with ambulatory care sensitive conditions (ACSCs), were more likely to be short, whereas admission of older patients and of those who arrived by ambulance, were more likely to be longer. [9]

In Israel there are 33 general public hospitals of which 11 are government hospitals, 9 are owned by the Clalit Health Fund, and the rest are public hospitals owned by others. Every resident is registered by law, with one of the health maintenance organizations (HMOs) which cover the expenses of all hospitalizations.

The rate of acute care beds in Israel at the end of 2022 was 1.75 per 1,000 people. This relatively low number of beds, reflects also on the number of beds in the medical wards, a fact that limits the capacity of admissions. Very short hospitalizations fill the inpatient wards and delay the transfer of patients from the ED to inpatient wards. Hospital managers in Israel, are searching for ways to decrease short hospitalizations as much as possible. Characterization of short hospitalizations may help to identify preventable hospitalizations and identify patients who should be hospitalized in short-stay units. In this study, we therefore decided to characterize the short hospitalizations in our large tertiary medical center.

## Materials and methods

The Sheba Medical Center (SMC) institutional review board (IRB) approved this retrospective study and waived Informed consent.

### Facility

This study was conducted at the SMC, Tel-Hashomer, Israel. This hospital is the largest governmental tertiary care academic medical center in the center of Israel with over 140,000 patients arriving at its ED annually. The hospital has over 1,900 beds, 7 medical wards with a total of 283 beds. More than 20,000 patients are hospitalized in medical wards every year, with an average length of stay during 2019 of 3.71 days and an occupancy rate of 90%.

### Study design

We conducted a retrospective analysis of consecutive adult patients admitted from January 1, 2013, to December 31, 2019. We limited the cohort to patients who were admitted to the medical wards from the ED. We excluded patients younger than 18, patients who were admitted to other wards, and patients who died in the hospital. All seven medical wards in the hospital were included in the study.

Short hospitalizations were defined as those lasting 0–1 calendar days, where 0 represents the admission date. This included any admission starting at 00:01 in the first day, and discharged before 24:00 in the following day.

We compared the ED features of the short hospitalizations with the non-short hospitalizations and with those who were discharged from the ED.

### Patient data

For each patient, we’ve retrieved ED information accumulated up-to the point of entry to the medical ward. The clinical parameters and the admission diagnosis presented in our study were sourced from the ED. These data included the *Admission date; Demographics*; *Arrival mode*; *Chief complaint*; *Previous ED visits* during the study time frame; *Previous hospitalizations* in our hospital during the study time frame; *Known comorbidities*: coded as International Classification of Diseases (ICD9) records and grouped using the diagnostic clinical classification software (CCS); *Vital signs in the ED* as were obtained upon contact with a triage nurse at the ED; *Emergency severity index* (ESI) measured at ED triage)subjective risk classification of patients, from 1 (most urgent) to 5 (least urgent), based on patients' acuity and resources needed. The ESI is the most widely used triage score. It is a subjective risk classification of patients assigned manually, usually by the triage nurse. The ESI relies heavily on provider judgment which can lead to inaccuracy and misclassification [10]. *Laboratory results* in the ED; *ED diagnoses* coded as ICD9 codes.

For each patient we’ve determined the ED shift at which he or she were admitted to the hospital. Three shifts were defined; morning (8:00–16:00), evening (16:00–24:00), and night (24:00–8:00).

Further, we evaluated each hospitalization according to the daily number of visits in the ED medical wing to reflect the daily load.

*Outcome measures;* revisit to our ED within 72 h from discharge and 30-days in and out of hospital mortality rate from presentation to the ED. The mortality data was retrieved from the ministry of interior of Israel.

### Analysis

Continuous features are reported as the mean and standard deviation (SD). Categorical elements are reported as percentages. Means of continuous variables were compared using the unpaired t-test for comparison between two groups and one-way Analysis of Variance (ANOVA) for comparison between more than two groups. Categorical variables were compared using the χ2 test. Spearman’s rank correlation was used to calculate correlations (r) between continuous variables.

A logistic regression analysis was conducted to examine which factors are independent predictors of short hospitalization. The features in the model are presented in Tables 1 and 2. Only items with less than 5% missing values were included in the regression model. The model enables to compute adjusted Odds Ratios (aOR), providing a measure of the strength of the associations in our data while adjusting for potential confounders. Additionally, using the model, we were able to calculate 95% Confidence Intervals (CI) for each aOR. To further ascertain the statistical significance of the associations, p-values were computed. Any value below the pre-determined alpha level of 0.05 was considered statistically significant.

**Table 1:** Comparison between non-short-term, short-term hospitalization and patients who were discharged from the emergency department

	Non short hospitalization (n = 73,132, 11.1%)	Short hospitalization (n = 59,994, 9.1%)	Discharged from ED (n = 524,302, 79.8%)	*P* value
*Demographics*				
Age, (years) mean (SD)	71 (16)	69 (17)	49 (21)	< 0.001
Female, N. (%)	33,695 (46)	28,581 (48)	261,953 (50)	< 0.001
Arrival mode				
Walk-in, N. (%)	36,007 (49)	32,560 (54)	437,616 (83)	< 0.001
Ambulance, N. (%)	35,234 (48)	25,869 (43)	77,629 (15)	< 0.001
Intensive care ambulance, N. (%)	1668 (2)	1443 (2)	2111 (0.4)	< 0.001
Unknown, N. (%)	223 (0.3)	122 (0.2)	1574 (0.3)	< 0.001
*Comorbidities*				
IHD, N. (%)	19,126 (26)	15,576 (26)	34,772 (7)	< 0.001
CHF, N. (%)	18,668 (25)	13,573 (23)	21,217 (4)	< 0.001
DM, N. (%)	23,818 (33)	17,444 (29)	43,208 (8)	< 0.001
CRF, N. (%)	10,721 (15)	6695 (11)	11,143 (2)	< 0.001
COPD, N. (%)	6782 (9)	4800 (8)	8513 (2)	< 0.001
Oncological patients, N. (%)	17,417 (24)	12,685 (21)	52,454 (10)	< 0.001
ESI, mean (SD)	2.9 (0.4)	3.0 (0.4)	3.3 (0.6)	< 0.001
*Laboratory tests*				
HGB (g/dl), mean (SD)	11.8 (2.2)	12.3 (2.1)	13.1 (1.8)	< 0.001
WBC (10^9^/L), mean (SD)	10.9 (9.4)	9.8 (7.7)	8.9 (4.5)	< 0.001
CRP (mg/L), mean (SD)	68.3 (83.8)	38.2 (62.3)	18.9 (37.2)	< 0.001
Creatinine (mg/dL), mean (SD)	1.4 (1.3)	1.2 (1.0)	0.9 (0.6)	< 0.001
Glucose (mg/dL), mean (SD)	152.1 (87.4)	143.4 (76.7)	117.0 (47.3)	< 0.001
*Outcome measures*				
72 h revisit to ED, N. (%)	3199 (4.4)	2224 (3.7)	28,690 (5.5)	< 0.001
30 days mortality, N. (%)	5144 (7.0)	2594 (4.3)	4844 (0.9)	< 0.001

The table presents the differences between the parameters of non-short and the short hospitalizations, and those who were discharged from the ED. *ED* Emergency department, *SD* Standard deviation, *IHD* ischemic heart disease, *CHF* Congestive heart failure, *DM* Diabetes mellitus, *CKD* chronic kidney disease, *COPD* Chronic obstructive pulmonary disease, *ESI* Emergency Severity index, *HGB* Hemoglobin, *WBC* White blood cells, CRP C reactive protein

**Table 2:** The main chief complaints in the short hospitalization group. The complaints are limited to those counting at least 1% of the entire cohort

Chief complaint	Number of visits with the complaint	Short hospitalization rate (%)
Suicide attempt	266	80
Hypertension	344	69
ENT complaint	147	62
Cardiac complaint	18,643	59
Neurological complaint	10,131	56
Anemia	940	55
Nausea	136	54
Hyperglycemia	283	53
Hypoglycemia	232	53
Syncope	2188	52

*ENT complaint* Ear nose and throat, *Cardiac complaint* chest pain, palpitations, arrhythmia, ECG changes, congestive heart failure, *Neurological complaint* dizziness, headache, seizures, minor ischemic event

Area under the receiver operating characteristic curve (AUC) metric was used to assess the ability of single variables to predict short hospitalizations. Each single variable AUC was calculated by using the variable as a single input, and short hospitalization as a target. Since some of the variables were categorical, we’ve used a gradient boosting model (CatBoost) to calculate the AUCs. Each model included 100 iterations, with a random data split of 80% training and 20% testing.

Bootstrapping validations (1000 bootstrap resamples) were used to calculate 95% confidence intervals (CI) for all metrics.

## Results

### Study cohort

**Fig. 1:**
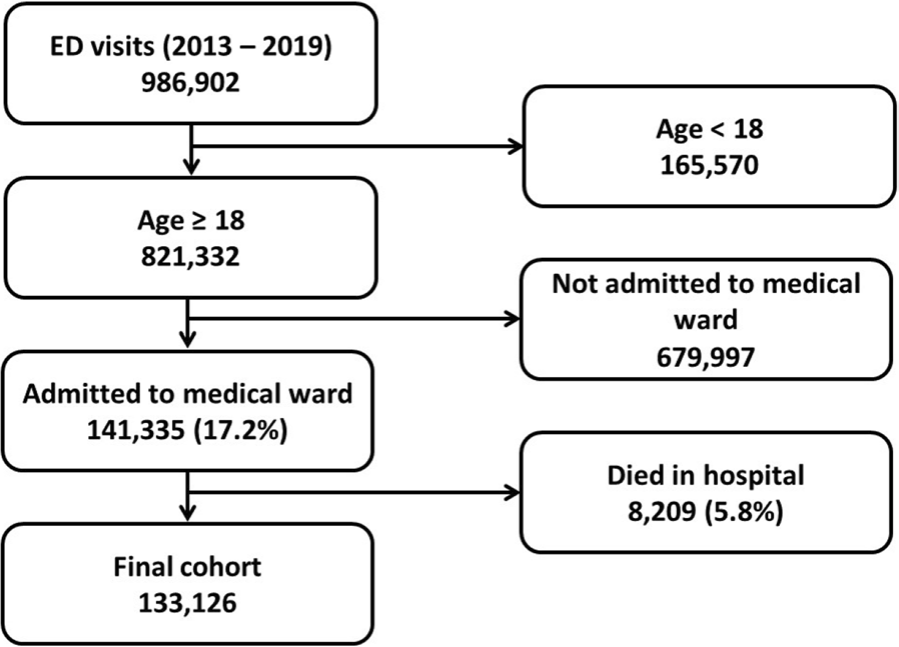
Study inclusion flowchart. *ED* emergency department

During the seven-year study, our ED recorded 986,902 visits. Of these, 821,332 were adult patients. 141,335 (17.2%) of the adult patients-visits were admitted to the medical wards. Of those 8,209 (5.8%) patients died in hospital. Thus, the final cohort included 133,126 patients -visits who were admitted to the medical wards. Figure 1 presents the study’s inclusion chart.

Most patients admitted to the medical wards stayed in the hospital less than 4 days. Overall, 59,994 (45.1%) short-term hospitalizations were identified and there were not seasonal differences.

### Cohort characteristics

Patients in the short hospitalization group were younger, were more likely to be females and had more previous visits in the ED and previous hospitalizations. They arrived less by an ambulance and had less comorbid diseases (Table 1). They had a higher emergency severity index (ESI), higher blood pressure (BP) levels (systolic BP 138 ± 27 vs. 134 ± 50 mmHg and diastolic BP 75 ± 16 vs. 73 ± 17 mmHg), higher saturation (95.9 ± 11.2 vs. 95.1 ± 41.2%), lower heart rate (84 ± 37 vs. 90 ± 49 beats/min), lower body temperature (36.9 ± 0.9 vs. 37.0 ± 1.1 ^0^C) and better laboratory parameters than those in the non-short hospitalization group (Table 1).

The 30-days mortality rate was 7.0% in the non-short hospitalization group, 4.3% in the short hospitalization group and 0.9% in those who were discharged from the ED (Table 1). The 72-h revisit rate was 4.4% in the non-short hospitalization group, 3.7% in the short hospitalization group and 5.5% in those who were discharged from the ED (Table 1). The overall 72-h readmission rate was 3.0%.

In the multivariable analysis, we included a comprehensive set of variables: age, gender, arrival mode, comorbidities (hypertension, congestive heart failure, diabetes mellitus, chronic kidney disease, chronic obstructive pulmonary disease, known malignancy), vital signs (systolic blood pressure, diastolic blood pressure, heart rate, temperature, oxygen saturation), Emergency Severity Index, and laboratory values (white blood cell count, blood urea nitrogen, creatinine, glucose). These variables were analyzed together in the model to predict the outcome of short hospitalizations. The following variables remained statistically significant as independent predictors of short-term hospitalization: Age (*p* < 0.001), Sex (*p* = 0.034), Arrival mode (*p* = 0.001), Ischemic Heart Disease (*p* < 0.001), Congestive Heart Failure (*p* = 0.002), Diabetes Mellitus (*p* = 0.008), Chronic Kidney Disease (*p* = 0.025), Emergency Severity Index (*p* < 0.001), Systolic Blood Pressure (*p* < 0.001), Diastolic Blood Pressure (*p* < 0.001), Heart Rate (*p* < 0.001), Temperature (*p* < 0.001), White Blood Cell count (*p* < 0.001), Creatinine (*p* < 0.001), and Glucose (*p* < 0.001). The variables Oxygen Saturation (*p* = 0.4) and Chronic Obstructive Pulmonary Disease (*p* = 0.115) did not achieve statistical significance and thus were not independent predictors of short-term hospitalization in our analysis.

Comparison of the hospitalized patients to those who were discharged shows that patients who were discharged from the ED, were younger, were more likely to be females, came less with ambulance and had less comorbidities. They had lower systolic and higher diastolic BP levels and they had better laboratory results. They had a lower 30-days mortality rate, but they have higher rate of revisit to our ED within 72 h (Table 1).

### Analysis of single variables association with short hospitalization rate

The main chief complaints and the ICD-9 ED diagnoses in the short hospitalization group are shown in Tables 2 and 3, respectively. The highest rate of chief complaint in the short hospitalization group was suicide attempt (80.0%), followed by hypertension (68.6%) (Table 2). However, these complaints accounted for only 449 hospitalizations (Table 2). The highest numbers of short hospitalizations were due to cardiac complaints (10,919 out of 18,643 visits) and neurological complaints (5674 out of 10,131 visits) (Table 2). The highest rate of ED diagnoses in the short hospitalization group was suicide attempt/self-inflicted poisoning (82.7%), followed by palpitations (69.9%) and vertigo (69.3%) (Table 3). Among patients with cardiac complaints, the rate of short hospitalization was very high in those with chest pain, palpitations and atrial fibrillation (Table 1S). Among patients with neurological complaints, the rate of short hospitalization was very high in those with transient ischemic attacks, convulsions, dizziness, headache and vertigo (Table 2S). Like the entire study group, those with neurological and cardiac complaints who were hospitalized for short term were younger, arrived less by an ambulance and had less comorbid diseases (Table 3S and 4S). Analysis of the effects of shifts on the rate of short-term hospitalizations revealed that the highest rate (58.4%) was during the night shift and the lowest rate (35.4%) was during the evening shift.

**Table 3:** The ten emergency department diagnoses (ICD-9 coded) with the highest short hospitalization rate. The diagnoses are limited to those counting at least 1% of the entire cohort

Diagnosis (ICD-9 coded)	Number of visits with the diagnosis	Short hospitalization rate (%)
SUICIDE/SELF INFICETED POISON UNSP DRUG/MED (E950.5)	359	83
PALPITATIONS (785.1)	336	70
VERTIGO -CEREB VAS LATE EFF (438.85)	437	69
TRANSIENT CEREBRAL ISCHEMIA (435.9)	1636	67
DIZZINESS AND GIDDINESS (780.4)	1212	65
PAROX ATRIAL TACHYCARDIA (427.0)	150	64
HEADACHE (784.0)	527	64
CHEST PAIN (786.5)	12,077	64
VERTBROBASLR ARTERY SYND (435.3)	136	63
CNS DISORDER NOS (349.9)	269	63
OTHER CONVULSIONS (780.39)	1168	62

The ED daily load positively correlated with the short hospitalization rate (r = 0.35, *p* < 0.001). This correlation was maintained after stratifying for individual week days.

The six single variables with the highest AUC for predicting short hospitalization is presented in Table 4. C-reactive protein (CRP) showed the overall highest AUC of 0.63. Other laboratory tests included elevated troponin (AUC 0.62) and low albumin (0.61). Among the non-laboratory tests the highest predictors included the ED diagnosis (AUC 0.62), followed by the ED shift (AUC 0.61) and chief complaint (AUC 0.60).

**Table 4:** The single variables with the highest area under the curve for predicting short hospitalization

	Not short vs. short hospitalizations	Area under the curve
CRP (mg/L)	68.3 ± 83.8 vs. 38.2 ± 62.3	0.63 (95% CI:0.62–0.63)
ED diagnosis		0.62 (95% CI:0.61–0.62)
Troponin-I (micg/L)	0.2 ± 1.5 vs. 0.1 ± 0.6	0.61 (95% CI:0.60–0.62)
Albumin –Blood (g/L)	3.6 ± 0.6 vs. 3.8 ± 0.6	0.61 (95% CI:0.60–0.62)
ED shift		0.60 (95% CI:0.60–0.61)
Chief complaint		0.60 (95% CI:0.59–0.61)

*CRP *C-reactive protein, *ED* emergency department

## Discussion

In the present study, we identified several characteristics of short hospitalizations. Cardiac and neurological complaints, made up 27.4% of the short hospitalizations. Young age and few comorbidities were associated with short hospitalizations. Night shifts and ED daily patient load were also associated with short hospitalizations. Patients in the short hospitalization group had a lower 30-days mortality rate than those in non-short hospitalization group, but they had higher mortality rate than the group of patients who were discharged from the ED. Of all hospitalizations during the study period, 45% were of short stay. This high rate can be explained by the criteria of short hospitalization, which included hospitalizations after midnight of the day before (making the maximal length of stay nearly 48 h, with a single overnight in-between).

Patients with short-term hospitalizations were typically younger and had fewer comorbidities. Their laboratory results also indicated that they were healthier since they had lower CRP and troponin and higher hemoglobin and albumin levels.

The most frequent complaints associated with a short hospitalization were suicide attempts and hypertension. Patients with suicide attempts are hospitalized for short psychiatric supervision and patients with hypertension probably do not require hospitalization. However, these complaints accounted for only 0.7% of the total number of short hospitalizations. We also found that the ED daily load determined the rate of short hospitalizations. Similarly, Chen et al. showed that increased ED occupancy was associated with higher hospital admission rates [11]. The effect of ED load on the rate of short hospitalizations can explain our findings that the day of admission also affected the rate of short hospitalizations. The day of the admission reflects a higher patient load on the ED, with Sunday and Monday usually being the busiest days of the week at the SMC. The high rate of short hospitalizations during the night shifts may also reflect the ED load since during these shifts, less physicians and nurses are on duty as compared with the day shifts. The effect of the admission week-day on outcome was described by Fehlmann et al., who showed that patients admitted in the general internal medicine division during week-ends, had different outcomes than those admitted on a week-day [12]. Similarly, Schmidt et al. showed that there is a considerable seasonal variability in the length of stay. [13]

The decision about the dispositions of patients is made by the attending physician of the ED. There are differences between the staffing of the ED: the proportion of seniors to residents is higher during day shifts and during the week as compared to night shifts and weekends. Our findings might suggest that reinforcement of staff in the busy hours or days, may reduce short hospitalization rates. Similar observation was described by Dawood et al., who demonstrated that reinforcement of ED, reduces acute admissions to medical department [14].

Reducing the rate of short hospitalizations to free up beds for more complex cases is crucial. Still, it is debatable whether or not short hospitalizations are inappropriate or preventable. Rizza et al. found that avoidable hospitalizations are more likely to have a shorter length of stay [15]. In contrast, Burgess et al. in New Zealand didn’t find that a short-stay in an acute general medical unit is necessarily an inappropriate admission [16]. Johnston J. et al. indicated that the most common diagnoses of preventable hospitalizations were congestive heart failure and chronic obstructive pulmonary disease [3]. These diagnoses were not found in our research. From our study we were unable to conclude whether short hospitalizations are preventable. Indeed, the 30-days mortality rate in the short hospitalization group was lower than in those with the non-short hospitalization group, but it was significantly higher than in the group of patients who were discharged from the ED. This may suggest that short hospitalizations were at least partially justified. However, even if short hospitalizations are appropriate, they pose a burden on the medical wards and therefore we should consider admitting these patients in a short-stay units (SSU's). There is a debate whether SSU's are beneficial for adults with internal medicine diseases and conditions [17]. Strom et al. concluded that SSU hospitalization was associated with a lower risk of adverse events, less functional decline, fewer readmissions, and shorter hospital stay [18]. SSU’s can reduce the rate of short hospitalizations in medical wards and ease the load on these departments. Choosing the appropriate patients for these units is crucial [19]. Our findings showed that most of the patients with short hospitalizations had cardiac complaints (18%) and neurological complaints (9.4%). Opening short stay units for these patients may be appropriate in-order to lower the rate of short hospitalizations in medical ward [20]. Cardiac short stay units are well accepted [21, 22], and can reduce the hospitalization cost and occupancy, whereas short-stay neurological units are less common. Recently, Paolucci M et al. described their experience with a neurological short stay unit during the COVID-19 pandemic [23]. Since many patients with neurological complaints were hospitalized for a short term, we believe that hospitalization of these patients in a neurological SSU may reduce the number of short hospitalizations and ease the burden on the medical wards. Opening a neurological SSU will enable a fast evaluation of these patients and prevent the load on the medical wards. It is clear that opening a neurological SSU will require additional nursing, physician staff and space. From our experience with cardiac SSU [22], we believe that despite the expenses of additional staff and space, opening a neurological SSU will be cost–benefit. The alternative of hospitalization in a waiting ward may be less appropriate for these patients since most of them need a short further evaluation.

The strength of our study is that the data are based on a large number of hospitalizations. The limitation of the study is that all the information was computerized and was based on the input of the attending physicians in the ED. Moreover, our study describes a single large tertiary center in Israel and therefore the results of the study are applied to a medical system with a large ED load and a shortage of in-hospital beds. In Israel, there are several large hospitals with a large ED load and we believe that they admit similar patients and are facing the same problems. In addition, we didn’t have the discharge diagnosis to confirm the admission diagnoses. However, our system is reliable and we believe the information is accurate. Another limitation is that we included only the first vital signs measured by the triage nurse. We did not include changes in vital signs. We also were unable to tell what percentage of the short hospitalizations were appropriate, and how many of them were preventable. However, our aim was to identify the causes of short hospitalizations and to suggest a way to lower the rate of short hospitalizations leading to a lower load on the medical wards. Furthermore, the exclusion of data on repeated visits limits the scope of our findings and future studies might benefit from employing mixed models or generalized estimating equation (GEE) to analyze these patterns more comprehensively.

## Conclusions

Cardiac and neurological complaints made up more than a quarter of the short hospitalizations. Short hospitalization in the medical ward is associated with young age and few comorbidities. Lowering the ED load may prevent some of these short hospitalizations. Opening SSUs for patients with cardiac and neurological problems may decrease the rate of short hospitalizations and improve the care of these patients and the care of patients in the medical wards.

## Supplementary Information

Additional file1

## Data Availability

The datasets during and/or analyzed during the current study available from the corresponding author on reasonable request**.**
