# Supplementary material for: Admission characteristics of patients with short term hospitalization
Source: Isr J Health Policy Res. 2024 Sep 26;13:51. doi: 10.1186/s13584-024-00639-3 (PMC11425996; doi:10.1186/s13584-024-00639-3)
Supplement: Supplementary file 1 — Additional file1 [file 13584_2024_639_MOESM1_ESM.docx]

Table 1S: The rate of short hospitalizations according to the most common cardiac diagnosis in those who presented to the emergency department with cardiac complains.

| **Percent of short hospitalization** | **Non short hospitalization** | **Short hospitalization** | **total** | **Diagnosis ( ICD 9 coded)** |
| --- | --- | --- | --- | --- |
| 65 | 3315 | 6055 | 9370 | Chest pain (786.5) |
| 58 | 513 | 717 | 1230 | Atrial fibrillation (427.31) |
| 54 | 303 | 358 | 661 | Atrial fibrillation/flutter (427.3) |
| 51 | 251 | 262 | 513 | Intermediate coronary syndrome (411.1) |
| 43 | 219 | 165 | 384 | Congestive heart failure(428) |
| 51 | 184 | 188 | 372 | Subendocardial infarct (410.71) |
| 72 | 68 | 172 | 240 | Palpitations (785.1) |

ICD9 = International Classification of Diseases

Table 2S: The rate of short hospitalizations according to the most common neurological diagnosis in those who presented to the emergency department with neurological complains.

| **Percent of short hospitalization** | **Non short hospitalization** | **Short hospitalization** | **total** | **Diagnosis ( ICD 9 coded)** |
| --- | --- | --- | --- | --- |
| 67 | 337 | 692 | 1029 | Transient cerebral ischemia (435) |
| 64 | 289 | 509 | 798 | Convulsions (780.39) |
| 47.3 | 532 | 479 | 1011 | Acute, but ill-defined, cerebrovascular disease (436) |
| 69 | 214 | 484 | 698 | Dizziness and giddiness (780.4) |
| 59 | 264 | 378 | 642 | Stroke (CVA) ischemic (434.91) |
| 38 | 243 | 149 | 392 | Acute or chronic cerebrovascular insufficiency (437.1) |
| 59 | 146 | 213 | 359 | Syncope and collapse (780.2) |
| 65 | 116 | 216 | 332 | Headache (784.0) |
| 71.2 | 72 | 178 | 250 | Vertigo (438.85) |

ICD9 = International Classification of Diseases, CVA = Cerebrovascular accident

Table 3S: Characteristics of patients with neurological complains in the short and non-short hospitalization groups

| Characteristics | Short hospitalization (n = 5674, 56.0%) | Non short hospitalization (n = 4457, 44.0%) | P value |
| --- | --- | --- | --- |
| Age, mean (SD), y | 68.0 (16.5) | 71.1 (14.6) | <0.001 |
| Female, N. (%) | 2800 (49.3) | 2110 (47.3) | 0.059 |
| **Arrival mode** |  |  |  |
| Walk-in, N. (%) | 2822 (49.7) | 1898 (42.6) | <0.001 |
| Ambulance, N. (%) | 2727 (48.1) | 2453 (55.0) | <0.001 |
| Intensive care ambulance, N. (%) | 116 (2.0) | 91 (2.0) | <0.001 |
| Unknown, N. (%) | 9 (0.2) | 15 (0.3) | <0.001 |
| **Clinical parameters** |  |  |  |
| SBP, mean (SD), mmHg | 145.7 (27.6) | 146.1 (29.1) | 0.418 |
| DBP, mean (SD), mmHg | 78.7 (14.1) | 77.5 (17.9) | <0.001 |
| Heart rate, mean (SD), b/min | 78.2 (19.2) | 80.4 (28.4) | <0.001 |
| Temperature, mean (SD), Celsius | 36.8 (0.4) | 36.8 (0.6) | <0.001 |
| Saturation, mean (SD), SaO2 | 96.9 (14.3) | 96.5 (15.0) | 0.234 |
| **Comorbidities** |  |  |  |
| IHD, N. (%) | 1057 (18.6) | 933 (20.9) | 0.004 |
| CHF, N. (%) | 806 (14.2) | 755 (16.9) | <0.001 |
| DM, N. (%) | 1539 (27.1) | 1359 (30.5) | <0.001 |
| CRF, N. (%) | 355 (6.3) | 403 (9.0) | <0.001 |
| COPD, N. (%) | 217 (3.8) | 216 (4.8) | 0.012 |
| Oncological patients, N. (%) | 990 (17.4) | 853 (19.1) | 0.029 |
| ESI, mean (SD) | 2.9 (0.4) | 2.8 (0.5) | <0.001 |
| **Laboratory tests** |  |  |  |
| HGB, mean (SD), gr/dL | 13.1 (1.7) | 12.7 (2.0) | <0.001 |
| WBC, mean (SD), counts | 8800 (4400) | 9500 (5600) | <0.001 |
| CRP, mean (SD), units | 13.6 (30.3) | 28.4 (55.5) | <0.001 |
| Cr, mean (SD), mg/dL | 1.0 (0.7) | 1.2 (1.0) | <0.001 |
| Glu, mean (SD), mg/dL | 135.9 (64.0) | 144.6 (79.1) | <0.001 |

SBP = Systolic blood pressure, DBP = Diastolic blood pressure, ED = Emergency department, IHD = Ischemic heart disease, CHF = Congestive heart failure, DM = Diabetes Mellitus, CRF = Chronic renal failure, COPD = Chronic obstructive pulmonary disease, ESI = Emergency severity index, HGB – Hemoglobin, WBC = White blood cells, CRP = C reactive protein, CR = Creatinine, Glu = Glucose,

Table 4S: Characteristics of patients with cardiac diagnosis in the short and non-short hospitalization groups

| Characteristics | Short hospitalization (n = 10919, 58.6%) | Non short hospitalization (n = 7724, 41.4%) | P value |
| --- | --- | --- | --- |
| Age, mean (SD), y | 68.2 (14.2) | 71.0 (13.4) | <0.001 |
| Female, N. (%) | 4514 (41.3) | 3102 (40.2) | 0.083 |
| **Arrival mode** |  |  |  |
| Walk-in, N. (%) | 5983 (54.8) | 3978 (51.5) | <0.001 |
| Ambulance, N. (%) | 4494 (41.2) | 3458 (44.8) | <0.001 |
| Intensive care ambulance, N. (%) | 429 (3.9) | 283 (3.7) | <0.001 |
| Unknown, N. (%) | 13 (0.1) | 5 (0.1) | <0.001 |
| **Clinical parameters** |  |  |  |
| SBP, mean (SD), mmHg | 140.8 (25.8) | 139.2 (25.6) | <0.001 |
| DBP, mean (SD), mmHg | 77.5 (15.3) | 75.6 (14.1) | <0.001 |
| Heart rate, mean (SD), b/min | 81.3 (25.6) | 84.0 (26.5) | <0.001 |
| Temperature, mean (SD), Celsius | 36.8 (0.8) | 36.8 (1.2) | 0.002 |
| Saturation, mean (SD), SaO2 | 96.7 (10.3) | 96.5 (16.2) | 0.338 |
| **Comorbidities** |  |  |  |
| IHD, N. (%) | 4393 (40.2) | 3444 (44.6) | <0.001 |
| CHF, N. (%) | 2746 (25.1) | 2397 (31.0) | <0.001 |
| DM, N. (%) | 2998 (27.5) | 2567 (33.2) | <0.001 |
| CRF, N. (%) | 915 (8.4) | 961 (12.4) | <0.001 |
| COPD, N. (%) | 569 (5.2) | 448 (5.8) | 0.081 |
| Oncological patients, N. (%) | 1477 (13.5) | 1219 (15.8) | <0.001 |
| ESI, mean (SD) | 3.0 (0.3) | 2.9 (0.3) | <0.001 |
| **Laboratory tests** |  |  |  |
| HGB, mean (SD), gr/dL | 13.0 (1.8) | 12.4 (2.1) | <0.001 |
| WBC, mean (SD), counts | 8700 (3900) | 9200 (5500) | <0.001 |
| CRP, mean (SD), units | 16.4 (36.9) | 30.0 (56.1) | <0.001 |
| Cr, mean (SD), mg/dL | 1.1 (0.8) | 1.3 (1.0) | <0.001 |
| Glu, mean (SD), mg/dL | 135.2 (62.3) | 145.2 (72.5) | <0.001 |

SBP = Systolic blood pressure, DBP = Diastolic blood pressure, ED = Emergency department, IHD = Ischemic heart disease, CHF = Congestive heart failure, DM = Diabetes Mellitus, CRF = Chronic renal failure, COPD = Chronic obstructive pulmonary disease, ESI = Emergency severity index, HGB – Hemoglobin, WBC = White blood cells, CRP = C reactive protein, CR = Creatinine, Glu = Glucose,
